# Beyond the workplace: abusive supervision and employees’ compulsive consumption behaviors

**DOI:** 10.3389/fpsyg.2026.1885985

**Published:** 2026-10-21

**Authors:** Min Wang, Yue Zhang, Yi Lang, Yuqi Liu

**Affiliations:** 1 International Business School, Beijing Foreign Studies University, Beijing, China; 2 School of Economics and Management, East China Normal University, Shanghai, China

**Keywords:** abusive supervision, compulsive buying, compulsive eating, ego depletion, pay satisfaction

## Abstract

**Introduction:**

Although abusive supervision has been widely examined in relation to workplace outcomes, less is known about how it may spill over into employees’ off-job consumption behaviors. Drawing on the process perspective of ego depletion theory, this study examines how and when abusive supervision is associated with two forms of compulsive consumption: compulsive buying and compulsive eating. We propose that abusive supervision contributes to ego depletion, conceptualized as a state of impaired self-regulatory functioning, characterized by reduced motivation to maintain restraint and heightened responsiveness to rewarding cues. Accordingly, we propose that abusive supervision increases employees’ tendencies toward compulsive buying and compulsive eating by impairing their self-regulatory capacity through ego depletion. Furthermore, we examine pay satisfaction as a boundary condition influencing the extent to which ego depletion translates into compulsive consumption behaviors.

**Methods:**

Using a three-wave survey design, we collected data from 445 full-time employees in China.

**Results:**

The results showed that abusive supervision was positively related to employees’ compulsive buying and compulsive eating via ego depletion. Moreover, pay satisfaction positively moderated the relationship between ego depletion and compulsive buying, as well as the indirect effect of abusive supervision on compulsive buying via ego depletion. In contrast, the moderating effect of pay satisfaction on compulsive eating and the corresponding moderated mediation effect were not significant.

**Discussion:**

These findings offer an incremental extension of the abusive supervision literature by linking workplace mistreatment to employees’ consumption-related self-regulatory failures, clarify the role of process-based ego depletion in explaining off-job spillover, and suggest that pay satisfaction may function as a behavior-specific boundary condition shaping employees’ behavioral responses under conditions of impaired self-regulation.

## 1 Introduction

Abusive supervision, defined as subordinates’ perceptions of sustained hostile verbal and nonverbal behaviors from supervisors, has been consistently identified as a pervasive and detrimental workplace stressor (Tepper, 2000; Tepper et al., 2017). A substantial body of research has documented its harmful consequences for employees’ work-related outcomes, including reduced performance, increased workplace deviance, and impaired attitudes and well-being (Bhattacharjee and Sarkar, 2024; Carlson et al., 2012; Tepper et al., 2007). Beyond the workplace, emerging evidence indicates that employees often cope with such stress through self-focused strategies, leading to maladaptive off-job consequences such as insomnia, work–family conflict, and maladaptive consumption-related behaviors (Hur and Shin, 2025; Liu et al., 2017). However, this literature remains fragmented and has largely emphasized outward-directed responses such as retaliation and deviance (Fischer et al., 2021), leaving less understood how abusive supervision may translate into inward-focused, consumption-related coping behaviors in daily life. In particular, employees who experience abusive supervision may not only direct negative reactions toward the organization or supervisor, but may also turn to immediately rewarding behaviors in their personal lives as a way of coping with negative affect and impaired self-regulation. This study therefore examines whether and how abusive supervision is associated with two forms of off-job compulsive consumption–compulsive buying and compulsive eating.

To explain this relationship, we draw on the contemporary process perspective of ego depletion (Hur and Shin, 2025; Inzlicht and Schmeichel, 2012). Traditionally, ego depletion has been conceptualized as a state in which self-regulatory resources become diminished following sustained self-control demands (Baumeister et al., 1998; Muraven and Baumeister, 2000). However, recent debates have questioned whether self-control operates through a finite resource, leading scholars to call for a more precise and process-based understanding of ego depletion (Friese et al., 2019; Inzlicht et al., 2014). From this perspective, ego depletion is better understood as a state of impaired self-regulatory functioning characterized by shifts in motivation and attention: individuals become less motivated to continue exerting restraint and more oriented toward immediate gratification, while also becoming less attentive to self-control cues and more responsive to reward-related cues. Applying this perspective to abusive supervision, we argue that employees exposed to hostile supervisory behavior must repeatedly regulate negative emotions, inhibit retaliatory impulses, and monitor interpersonal threats. These sustained regulatory demands may impair employees’ self-regulatory functioning, making them more susceptible to compulsive consumption behaviors that offer immediate relief, comfort, or gratification.

Consistent with this perspective, we propose that ego depletion mediates the relationship between abusive supervision and two representative forms of compulsive consumption: compulsive buying and compulsive eating. We focus on these outcomes not merely as additional nonwork consequences of abusive supervision, but because they closely reflect the process-based understanding of ego depletion. According to this model, prior regulatory demands can impair subsequent self-regulation by shifting individuals’ motivation away from continued restraint and toward immediate rewards (Inzlicht et al., 2014; Inzlicht and Schmeichel, 2012). Compulsive buying and compulsive eating are especially relevant as both involve recurrent difficulties resisting immediately rewarding consumption urges despite awareness of potential negative consequences. As noted in a foundational framework for understanding dysfunctional consumption, Faber et al. (1995) identified compulsive buying and binge eating (i.e., compulsive eating) as two primary and interrelated forms of compulsive consumption, while also capturing distinct domains of daily life–material versus food-related consumption. Unlike general maladaptive behaviors, these behaviors further reflect a failure of sustained self-regulation, as individuals continue consumption despite awareness of its negative consequences. They therefore provide a concrete manifestation of consumption-related self-regulatory failure, allowing us to examine how abusive supervision spills over into employees’ personal lives through depleted self-regulation (Wu et al., 2012; Zhu et al., 2023).

Furthermore, we examine pay satisfaction as a critical boundary condition that shapes the extent to which ego depletion translates into compulsive consumption behaviors. Pay satisfaction is defined as “amount of overall positive or negative affect (or feelings) that individuals have toward their pay” (Miceli and Lane, 1991). Although pay satisfaction is generally viewed as a favorable work attitude, its role may depend on the behavioral context in which employees respond to impaired self-regulation. Drawing on the process model of ego depletion, depleted individuals are less motivated to maintain restraint and more oriented toward immediate gratification; they may also become less attentive to self-control cues and more responsive to reward-related cues (Inzlicht et al., 2014; Inzlicht and Schmeichel, 2012). However, the enactment of specific compulsive behaviors may depend on distinct proximal conditions. For compulsive buying, which inherently involves a monetary transaction, higher pay satisfaction may reduce perceived financial constraints and increase perceived financial latitude, making buying impulses more behaviorally feasible (Beatty and Ferrell, 1998; Hamilton et al., 2019). By contrast, compulsive eating may be less directly contingent on discretionary financial resources and more proximally driven by somatic cravings, visceral affect regulation, physiological cues, and habitual responses (Heatherton and Baumeister, 1991; Wood and Neal, 2007; Tiffany and Wray, 2012). Thus, rather than treating pay satisfaction as a general amplifier of compulsive consumption, we conceptualize it as a financially relevant boundary condition whose moderating role should be stronger for material coping behaviors such as compulsive buying than for somatic or habit-based behaviors such as compulsive eating.

Our study makes several theoretical contributions. First, we advance the abusive supervision literature by offering a process-based explanation of how abusive supervision spills over into employees’ personal lives. Departing from a traditional resource-depletion view, we draw on the contemporary process model of ego depletion, and explain not only whether abusive supervision affects employees beyond work, but also why such spillover may manifest as compulsive buying and compulsive eating as forms of consumption-related self-regulatory failure. Second, we contribute to the compulsive consumption literature by identifying abusive supervision as a workplace-related antecedent of compulsive buying and compulsive eating. Existing research has primarily focused on individual traits, emotional factors, and consumer-related influences. Our findings suggest that workplace mistreatment may also be relevant to understanding compulsive consumption through impaired self-regulation. Third, we reveal pay satisfaction as a behavior-specific boundary condition in the spillover of abusive supervision. Rather than viewing pay satisfaction as a general amplifier of compulsive consumption, our findings suggest that its role depends on whether the financial latitude it represents matches the behavioral requirements of the outcome. Specifically, pay satisfaction is more closely aligned with compulsive buying, which involves discretionary spending, than with compulsive eating, which is more proximally driven by somatic cravings, affect regulation, physiological cues, and habitual responses. By showing that pay satisfaction selectively strengthens the ego depletion–compulsive buying pathway, our study adds depth to the abusive supervision spillover literature and highlights the behavior-specific contingencies through which workplace mistreatment extends into employees’ nonwork lives.

## 2 Theory and hypotheses

### 2.1 Abusive supervision and subordinates’ ego depletion

Contemporary perspectives on ego depletion conceptualize it as a state of impaired self-regulation that emerges following sustained regulatory demands, rather than as the exhaustion of a finite psychological resource (Friese et al., 2019; Inzlicht and Schmeichel, 2012). From this perspective, prior acts of self-control undermine subsequent self-regulation by shifting individuals’ motivation away from continued restraint and toward immediate gratification, and by redirecting attention away from cues signaling the need for control and toward cues associated with reward or relief (Inzlicht et al., 2014). Accordingly, workplace stressors that require continuous self-regulation may contribute to heightened ego depletion by undermining individuals’ self-regulatory functioning.

We argue that abusive supervision contributes to subordinates’ ego depletion through two primary self-regulatory pathways. First, abusive supervision creates psychologically demanding interpersonal situations that require continuous self-regulation. As a sustained form of hostile supervisory behavior (Tepper, 2000), abusive supervision exposes subordinates to frequent negative treatment, which triggers strong emotional and behavioral reactions. However, due to hierarchical constraints and workplace norms, subordinates are often unable to express these reactions freely (Huang et al., 2023; Martinko et al., 2013). Instead, they must suppress negative emotions, inhibit retaliatory urges, and maintain appropriate workplace conduct. Sustained engagement in these regulatory activities may gradually reduce individuals’ willingness and ability to exert further self-control, thereby increasing susceptibility to self-regulatory impairment (Inzlicht et al., 2014; McAllister et al., 2018).

Second, abusive supervision generates a pervasive sense of threat and uncertainty that places additional demands on self-regulation. Specifically, experiencing abuse from a supervisor signals potential risks to one’s status, job security, and future within the organization (Li et al., 2022). As a result, subordinates may become increasingly vigilant and engage in repetitive thoughts concerning potential future mistreatment, the unpredictability of supervisory behavior, and their standing within the organization (Hur and Shin, 2025). Managing these concerns requires ongoing cognitive and emotional regulation, including monitoring potential threats, controlling anxiety, and adapting behavior to uncertain circumstances (Lazarus and Folkman, 1984). These processes may draw attention toward threat-related cues at work while reducing attention to cues that normally support self-control. Consistent with the process model of ego depletion, such attentional disruption may make employees less able or willing to recognize when self-control is needed and more responsive to cues associated with immediate relief or reward.

Taken together, abusive supervision increases the need for sustained emotional, behavioral, and cognitive regulation. Rather than suggesting that abusive supervision simply exhausts a finite psychological resource, we argue that repeated exposure to abusive supervision undermines subordinates’ self-regulatory functioning by altering their motivation and attention. Specifically, subordinates may become less motivated to continue exerting restraint, less attentive to self-control cues, and more oriented toward immediate relief or gratification. Consistent with the process perspective on ego depletion, prolonged exposure to these regulatory demands may undermine subordinates’ self-regulatory functioning, resulting in heightened ego depletion.

*H1: Abusive supervision is positively related to subordinates’ ego depletion.*

### 2.2 The mediation role of ego depletion between abusive supervision and subordinates’ compulsive buying and compulsive eating

Building on the process perspective of ego depletion theory, we propose that ego depletion increases compulsive buying and compulsive eating by impairing individuals’ self-regulatory functioning. Rather than reflecting the exhaustion of a finite resource, ego depletion represents a state in which individuals become less motivated to continue exerting restraint and more oriented toward immediate gratification after sustained regulatory demands (Inzlicht et al., 2014; Inzlicht and Schmeichel, 2012). At the same time, depleted individuals may become less attentive to cues that normally signal the need for self-control, such as long-term goals, behavioral standards, or potential negative consequences, and more responsive to reward-related cues. Consequently, they become more vulnerable to immediately rewarding behaviors that offer temporary emotional relief or gratification.

This process is particularly relevant to compulsive consumption behaviors. Compulsive buying and compulsive eating are characterized by recurrent difficulties in controlling consumption urges despite awareness of their negative consequences (Faber et al., 1995; Kakoschke et al., 2019). Unlike impulsive consumption, which often reflects spontaneous reactions to immediate situational cues, compulsive consumption represents a more persistent pattern of dysregulated behavior that is closely tied to self-regulatory difficulties and emotion-regulation motives (Black, 2007; Moulding et al., 2017; Verplanken and Sato, 2011). Thus, when ego depletion shifts individuals’ motivation away from restraint and toward gratification, while also redirecting attention toward rewarding consumption cues (Friese et al., 2019), individuals may become more vulnerable to using buying or eating as a means of short-term affect regulation.

In off-work settings, where organizational controls and behavioral constraints are relatively weak, employees experiencing ego depletion may be especially susceptible to easily accessible forms of gratification such as shopping and eating. Prior research suggests that work-related stressors and reduced self-regulatory capacity increase unhealthy eating and mood-driven consumption behaviors (Liu et al., 2017). These behaviors may provide temporary relief from negative affect or psychological discomfort, but repeated reliance on them can reinforce maladaptive consumption patterns over time (Dittmar et al., 2007; Tice and Bratslavsky, 2000). Therefore, ego depletion may increase the likelihood that employees engage in compulsive buying and compulsive eating as consumption-related manifestations of impaired self-regulation.

*H2a: Subordinates’ ego depletion is positively related to compulsive buying.*

*H2b: Subordinates’ ego depletion is positively related to compulsive eating.*

Building on the above arguments, we further propose a mediating role of ego depletion in linking abusive supervision to compulsive behaviors. As discussed, abusive supervision requires sustained emotional, behavioral, and cognitive regulation while simultaneously generating threat-related concerns and uncertainty. In turn, individuals experiencing ego depletion become more susceptible to immediately rewarding consumption behaviors and less capable of regulating consumption urges, making them more likely to engage in compulsive buying and compulsive eating. Therefore, ego depletion serves as a key mechanism through which abusive supervision spills over into employees’ compulsive consumption behaviors.

*H3a: Ego depletion mediates the relationship between abusive supervision and compulsive buying.*

*H3b: Ego depletion mediates the relationship between abusive supervision and compulsive eating.*

### 2.3 The moderating role of pay satisfaction

Pay satisfaction represents employees’ affective evaluation of their compensation (Miceli and Lane, 1991; Panaccio et al., 2014). Traditionally, pay satisfaction has been associated with positive workplace attitudes and outcomes. In the present study, we do not suggest that pay satisfaction directly produces compulsive consumption or that it generally undermines employee well-being. We propose that its moderating relevance depends on whether financial latitude constitutes an important behavioral condition for translating impaired self-regulation into a particular form of compulsive consumption.

Drawing on the process model of ego depletion, depleted individuals are less motivated to continue exerting restraint and more oriented toward immediate gratification; they may also become less attentive to self-control cues and more responsive to reward-related cues (Inzlicht and Schmeichel, 2012; Friese et al., 2019). Under such conditions, hedonic consumption impulses are already activated by regulatory fatigue. However, the behavioral expression of impaired self-regulation may also depend on behavior-specific opportunities and constraints. Thus, although compulsive buying and compulsive eating share a common self-regulatory basis, the conditions that enable these impulses to be enacted may differ across the two behaviors.

Pay satisfaction may strengthen the relationship between ego depletion and compulsive buying, because it reflects employees’ favorable evaluation of their compensation and may be associated with greater perceived financial latitude (Miceli and Lane, 1991; Williams et al., 2006). Purchasing is inherently a monetary transaction and therefore depends on individuals’ perceived ability to spend. Consumer research similarly suggests that perceived money availability and financial constraints influence whether consumption urges are translated into actual purchasing behavior (Beatty and Ferrell, 1998; Hamilton et al., 2019). Accordingly, when ego depletion weakens restraint and heightens orientation toward immediate rewards, higher pay satisfaction may make compulsive buying more behaviorally feasible. Conversely, employees with lower pay satisfaction may experience similar reward-seeking impulses when depleted, yet perceived monetary constraints may inhibit their enactment through purchasing. Therefore, we hypothesize that pay satisfaction positively moderates the relationship between ego depletion and compulsive buying behaviors.

*H4a: Subordinates’ pay satisfaction positively moderates the relationship between ego depletion and compulsive buying.*

The moderating relevance of pay satisfaction may be less direct for compulsive eating. Although compulsive eating also reflects impaired self-regulation, its enactment is not inherently contingent on substantial discretionary spending. Instead, eating behavior is more proximally shaped by food cravings, physiological and visceral cues, affect-regulation motives, and habitual responses (Heatherton and Baumeister, 1991; Wood and Neal, 2007; Tiffany and Wray, 2012). Consequently, the shift toward immediate gratification associated with ego depletion may translate into compulsive eating primarily through these somatic and affective processes rather than through perceived financial latitude. Pay satisfaction may therefore be less behaviorally matched to the mechanisms governing whether depleted individuals act on eating urges.

Nevertheless, because food consumption still entails some degree of economic access and because greater perceived financial latitude may broaden access to rewarding consumption opportunities, we examine whether pay satisfaction also strengthens the relationship between ego depletion and compulsive eating. We expect, however, that this moderating role should be theoretically weaker than for compulsive buying because financial latitude is less proximal to the enactment of compulsive eating.

*H4b: Subordinates’ pay satisfaction positively moderates the relationship between ego depletion and compulsive eating, although this moderating relationship is expected to be weaker than that for compulsive buying.*

Building on the process perspective of ego depletion, we propose a moderated mediation model whereby pay satisfaction shapes the extent to which impaired self-regulation is translated into compulsive consumption behaviors. Ego depletion may shift individuals’ motivation away from continued restraint and toward immediate gratification, while redirecting attention away from self-control cues and toward reward-related cues. However, whether such self-regulatory impairment ultimately manifests in compulsive buying or compulsive eating may depend on contextual factors that influence the feasibility of acting on consumption-related impulses.

Pay satisfaction reflects employees’ favorable evaluation of their compensation and overall rewards received from work (Panaccio et al., 2014). When employees are highly satisfied with their pay, they may perceive greater financial latitude and fewer tangible monetary constraints when facing consumption opportunities. Pay satisfaction should be particularly relevant to the indirect pathway leading to compulsive buying. Abusive supervision contributes to ego depletion, which increases vulnerability to immediately rewarding consumption; higher pay satisfaction may then facilitate the behavioral enactment of this vulnerability through purchasing by reducing perceived financial constraints. Thus, the indirect effect of abusive supervision on compulsive buying through ego depletion should be stronger when pay satisfaction is higher.

For compulsive eating, however, the corresponding conditional indirect effect may be less sensitive to pay satisfaction because the enactment of eating urges depends more proximally on somatic, affective, and habitual processes than on financial latitude. Although greater financial latitude may still facilitate access to rewarding food consumption, it is less central to the behavioral enactment of compulsive eating than to purchasing. We therefore examine the corresponding conditional indirect effect while recognizing that pay satisfaction represents a theoretically less proximal boundary condition for compulsive eating.

*H5a: Subordinates’ pay satisfaction positively moderates the relationship between abusive supervision and compulsive buying via ego depletion.*

*H5b: Subordinates’ pay satisfaction positively moderates the relationship between abusive supervision and compulsive eating via ego depletion, although this moderating relationship is expected to be weaker than that for compulsive buying.*

The theoretical model developed in this study is illustrated in Figure 1.

**FIGURE 1:**
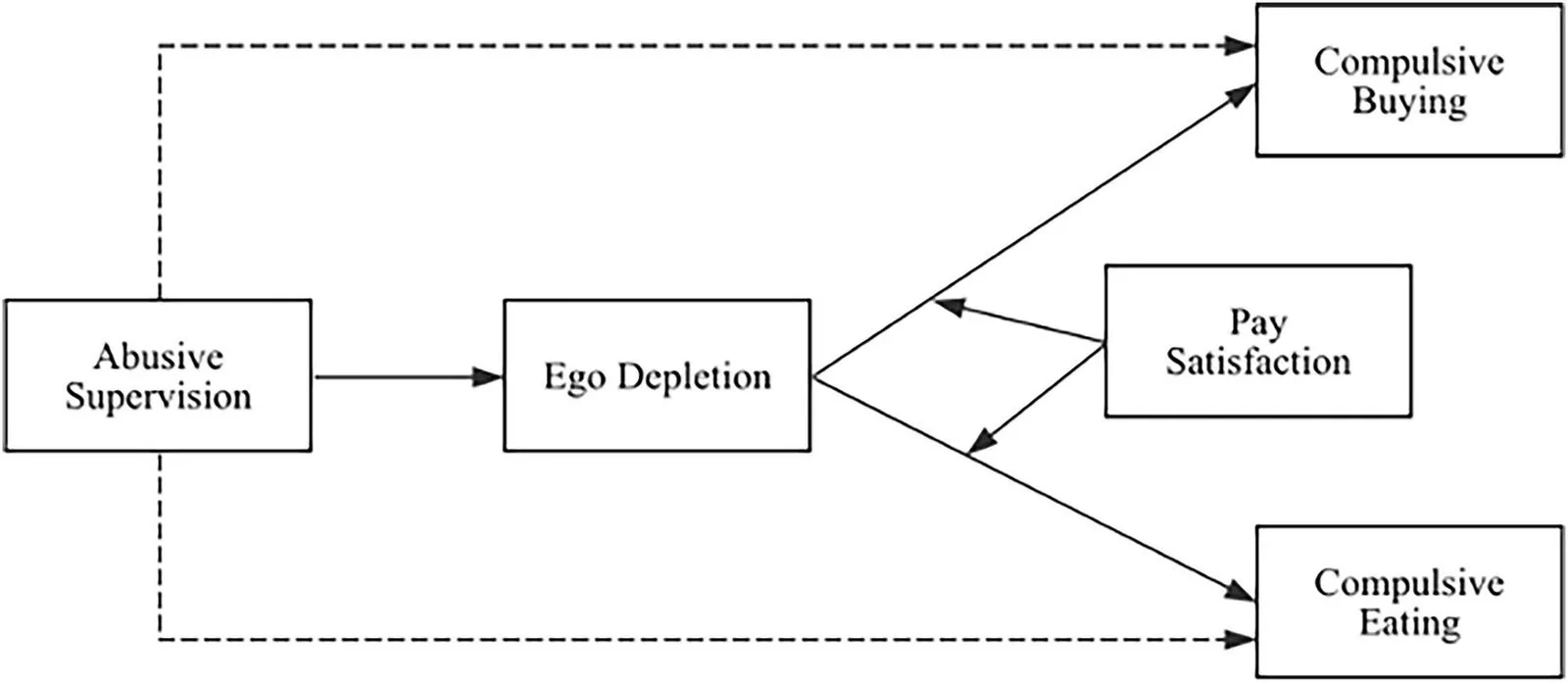
Theoretical model.

## 3 Methodology

### 3.1 Sample and procedure

Data for this study were collected via Credamo (a Chinese professional survey platform recognized by top international journals; Jin et al., 2021). The survey sample was restricted to full-time employees with work experience. We did not adopt a leader–member dyadic design because such designs typically require supervisors to evaluate employees’ behaviors in the workplace. However, the focal constructs in this study–compulsive buying and compulsive eating–are off-job consumption behaviors that occur in employees’ private lives and involve personal experiences that are not directly observable by supervisors or other external parties. Therefore, self-reports were deemed the most appropriate approach for capturing these constructs.

To mitigate potential common method bias, data were collected at three time points with a one-week interval between consecutive waves. This interval was chosen to ensure temporal separation while preserving theoretical proximity among abusive supervision, ego depletion, and subsequent off-job consumption behaviors. Specifically, the effects of impaired self-regulation may not be expressed immediately, as behaviors such as compulsive buying often depend on the availability of consumption opportunities (Beatty and Ferrell, 1998; Park et al., 1989), and compulsive eating may occur in non-work settings where emotional, physiological, and habitual cues become salient (Heatherton and Baumeister, 1991; Macht, 2008). However, an excessively long interval may introduce additional intervening experiences and weaken the linkage between abusive supervision and its downstream consequences. Thus, a one-week interval was considered appropriate for capturing the proposed spillover process. This choice is consistent with methodological work suggesting that longer lags are not necessarily preferable and that time lags should match the temporal characteristics of the focal process (Dormann and Griffin, 2015), as well as prior abusive supervision research using one-week intervals to examine mediating mechanisms and behavioral outcomes (Lian et al., 2012).

Specifically, at Time 1 (Week 1), participants reported demographic information and evaluated their immediate supervisor’s abusive supervision. A total of 700 valid questionnaires were collected at this stage. At Time 2 (Week 2), participants who had validly completed the first survey were invited to complete a second questionnaire, reporting pay satisfaction and perceived ego depletion. A total of 516 valid questionnaires were collected at this stage. At Time 3 (Week 3), participants who had validly completed the second survey were invited to complete a third questionnaire, reporting compulsive buying and compulsive eating. Following the three waves of data collection, the data were matched using unique user IDs. After removing invalid samples, a final sample of 445 valid matched responses was obtained. In addition, the temporal separation of the predictor, mediator, moderator, and outcome variables across different waves helped further mitigate common method bias by reducing consistency motives and respondents’ reliance on implicit theories.

The demographic profile of the sample was as follows: 59% were female; the largest age group was 26–35 years old, accounting for 60% of the sample; 84% held a bachelor’s degree or above. Each participant received approximately RMB 15 (approximately 2.1 US dollars) as compensation.

### 3.2 Measurement

All scales used in this study were adopted from well-established instruments. The questionnaire utilized a 7-point Likert scale, where responses ranged from 1 (“strongly disagree”) to 7 (“strongly agree”).

#### 3.2.1 Abusive supervision

We measured abusive supervision using the 15-item scale developed by Tepper (2000). Sample items include “My supervisor ridicules me.” The Cronbach’s alpha coefficient for this scale was 0.925.

#### 3.2.2 Ego depletion

We measured ego depletion using the 5-item scale developed by Lin and Johnson (2015). A sample item is “My mental energy is running low.” The Cronbach’s alpha coefficient for this scale was 0.925.

#### 3.2.3 Compulsive buying

We measured compulsive buying using the 13-item scale developed by Edwards (1993). Sample items include “I buy things even when I don’t need anything.” The Cronbach’s alpha coefficient for this scale was 0.961.

#### 3.2.4 Compulsive eating

We measured compulsive eating using the 15-item scale developed by Ruddock et al. (2017). A sample item is “I continue to eat despite feeling full.” The Cronbach’s alpha coefficient for this scale was 0.958.

#### 3.2.5 Pay satisfaction

We assessed pay satisfaction via a 9-item measurement scale adapted from Heneman and Schwab (1985). Respondents evaluated each item on a 7-point Likert scale, with anchors ranging from 1 (very dissatisfied) to 7 (very satisfied). Specific evaluated facets included employee benefit packages as a representative example. The Cronbach’s alpha coefficient for this scale was 0.940.

#### 3.2.6 Control variables

Based on prior research, we controlled for several demographic variables, including gender (1 = male and 2 = female), age (1 = under 18 years old, 2 = 18–25 years old, 3 = 26–35 years old, 4 = 36–45 years old, 5 = 46–55 years old, and 6 = over 55 years old), organizational tenure (1 = less than 1 year, 2 = 1–3 years, 3 = 4–6 years, 4 = 7–10 years, and 5 = more than 10 years), education level (1 = high school or below, 2 = junior college, 3 = bachelor’s degree, and 4 = graduate degree or above), and monthly income (1 = RMB 1,500 or below, 2 = RMB 1,501–3,000, 3 = RMB 3,001–5,000, 4 = RMB 5,001–8,000, 5 = RMB 8,001–10,000, and 6 = RMB 10,001 or above). Monthly income was included to distinguish the subjective evaluation captured by pay satisfaction from employees’ objective compensation level. Consistent with recommendations by Bernerth and Aguinis (2016), age and organizational tenure were not simultaneously included in the models due to their high correlation (*r* = 0.697), which raises potential multicollinearity concerns (O’Brien, 2007). Therefore, we retained age as the primary control variable in the main analyses. As a robustness check, we re-estimated all models by replacing age with organizational tenure, and the substantive pattern of results remained unchanged.

### 3.3 Statistical analysis

We performed statistical analyses on the collected data. The specific analytical procedures were as follows: First, we assessed potential common method bias using Harman’s single-factor test and an unmeasured latent method factor (ULMC) analysis. Second, we conducted confirmatory factor analyses (CFA) on the five variables. Third, we performed descriptive statistics, correlation analyses, and regression analyses to test the hypotheses. Finally, we utilized an integrated latent variable structural equation model to test the overall moderated mediation model.

## 4 Results

### 4.1 Common method bias and confirmatory factor analysis

To mitigate the potential influence of common method bias, we adopted a multi-wave measurement design in the study. In addition, we conducted Harman’s single-factor test. The results showed that the first unrotated factor accounted for 34.638% of the total variance, which was below the recommended threshold of 40%, suggesting that common method variance was unlikely to be a serious concern.

To provide a more rigorous assessment beyond Harman’s single-factor test, we further conducted an unmeasured latent method factor (ULMC) analysis by adding a latent method factor to the hypothesized five-factor measurement model. As shown in Table 1, compared with the original five-factor model (CFI = 0.910, RMSEA = 0.049), the inclusion of the latent method factor resulted in only modest improvements in model fit (CFI = 0.920, RMSEA = 0.046; ΔCFI = 0.010, ΔRMSEA = 0.003). These findings suggest that common method variance is unlikely to substantially account for the observed relationships.

**TABLE 1:** Measurement model comparisons (*N* = 445).

Model	χ^2^	*df*	χ^2^/*df*	RMSEA	SRMR	CFI	TLI
1. Five-factor model (AS; ED; PS; CB; CE)	3124.363	1525	2.049	0.049	0.056	0.910	0.906
2. Five-factor model + ULMC	2888.552	1478	1.954	0.046	0.067	0.920	0.914
3. Four-factor model (AS+ED; PS; CB; CE)	4234.987	1529	2.770	0.063	0.082	0.847	0.841
4. Three-factor model (AS+ED+PS; CB; CE)	5592.973	1532	3.651	0.077	0.089	0.771	0.761
5. Two-factor model (AS+ED+PS; CB+CE)	8220.378	1534	5.359	0.099	0.120	0.623	0.608
6. One-factor model (AS+ED+PS+CB+CE)	10743.854	1535	6.999	0.116	0.144	0.481	0.460

AS, abusive supervision; ED, ego depletion; PS, pay satisfaction; CB, compulsive buying; CE, compulsive eating. “+” indicates factor combination; ULMC, unmeasured latent method construct. The ULMC model was estimated by adding a latent method factor to the hypothesized five-factor measurement model.

We conducted confirmatory factor analyses (CFA) to examine the discriminant validity of abusive supervision, ego depletion, compulsive buying, compulsive eating, and pay satisfaction. As shown in Table 1, the five-factor model exhibited the best fit to the data (χ^2^ = 3124.363, *df* = 1525, χ^2^/*df* = 2.049, RMSEA = 0.049, SRMR = 0.056, CFI = 0.910, TLI = 0.906) and fit the data significantly better than all alternative models. These results provide evidence of adequate discriminant validity among the five focal constructs.

### 4.2 Descriptive statistics

The results of the descriptive statistics and correlation analysis are presented in Table 2. The results indicate that abusive supervision was significantly positively correlated with ego depletion (*r* = 0.504, *p* < 0.01), ego depletion was significantly positively correlated with compulsive buying (*r* = 0.357, *p* < 0.01) and compulsive eating (*r* = 0.560, *p* < 0.01), abusive supervision was significantly positively correlated with compulsive buying (*r* = 0.293, *p* < 0.01), and abusive supervision was significantly positively correlated with compulsive eating (*r* = 0.395, *p* < 0.01). These results provide preliminary support for the basic hypotheses of this study.

**TABLE 2:** Descriptive statistics and correlations (*N* = 445).

Variables	Means	S.D.	1	2	3	4	5	6	7	8	9
1 Gender	1.587	0.493	−0.076	−0.139**	0.311**	0.245**	−0.104*	0.504**	−0.625**	−0.208**	0.539**
2 Age	2.998	0.746									
3 Education	2.930	0.673	0.096*								
4 Monthly income	4.393	1.155	−0.090	0.150**							
5 Tenure	2.791	1.073	−0.125**	0.697**	−0.058						
6 Abusive supervision	2.219	0.838	−0.007	−0.059	−0.014	−0.097*					
7 Ego depletion	2.919	1.422	0.075	−0.264**	−0.014	−0.269**	−0.328**				
8 Pay satisfaction	4.727	1.250	−0.06	0.128**	0.109*	0.330**	0.232**	−0.421**			
9 Compulsive buying	3.257	1.462	0.114*	−0.127**	−0.006	−0.221**	−0.121*	0.293**	0.357**		
10 Compulsive eating	3.044	1.278	0.111*	−0.216**	−0.063	−0.237**	−0.240**	0.395**	0.560**	−0.444**	

*p < 0.05 (two-tailed),

**p < 0.01 (two-tailed).

### 4.3 Hypothesis testing

#### 4.3.1 Direct effects

As demonstrated in Table 3, after entering all control variables, abusive supervision was significantly positively related to ego depletion (*b* = 0.804, *SE* = 0.066, *p* < 0.001); ego depletion was significantly positively related to compulsive buying (*b* = 0.319, *SE* = 0.048, *p* < 0.001), and also to compulsive eating (*b* = 0.466, *SE* = 0.038, *p* < 0.001). Therefore, Hypotheses 1 and 2 were supported.

**TABLE 3:** Direct effects (*N* = 445).

Variables	Ego depletion	Compulsive buying	Compulsive eating
	Model 1	Model 2	Model 3
Constant	3.011***	2.624***	2.382***
Gender	0.125	0.221	0.171
Age	−0.382***	−0.028	−0.123
Education	0.046	0.070	−0.098
Monthly income	−0.242***	−0.175**	−0.071
Abusive supervision	0.804***		
Ego depletion		0.319***	0.466***
*R* ^2^	0.348	0.152	0.331
*F*	46.861***	15.677***	43.519***

**p < 0.01,

***p < 0.001.

#### 4.3.2 Mediating effects

We used the PROCESS macro (Model 4) to test the mediating effects of ego depletion using the Bootstrap method, controlling for gender, age, education level, and monthly income. As shown in Table 4, based on 5,000 bootstrap resamples, the indirect effect of abusive supervision on compulsive buying via ego depletion was 0.183, and the 95% bias-corrected confidence interval did not contain zero (LLCI = 0.082, ULCI = 0.302). Hypothesis 3a was supported.

**TABLE 4:** Bootstrap tests of the mediating effects of ego depletion (*N* = 445).

Effect type	Effect estimate	*SE*	95%LLCI	95%ULCI
Total Effect (Y_1_)	0.474	0.078	0.321	0.626
Direct effect (X→Y_1_)	0.291	0.088	0.118	0.465
Indirect effect (X→M→Y_1_)	0.183	0.056	0.082	0.302
Total effect (Y_2_)	0.565	0.064	0.439	0.691
Direct effect (X→Y_2_)	0.255	0.068	0.121	0.389
Indirect effect (X→M→Y_2_)	0.310	0.053	0.213	0.420

X, abusive supervision; M, ego depletion; Y_1_, compulsive buying; Y_2_, compulsive eating.

Using the same method, we examined the mediating role of ego depletion in the relationship between abusive supervision and compulsive eating. The results are presented in Table 4. The indirect effect of abusive supervision on compulsive eating via ego depletion was 0.310, and the 95% bias-corrected confidence interval did not contain zero (LLCI = 0.213, ULCI = 0.420). Hypothesis 3b was supported.

#### 4.3.3 Moderating effects

Prior to testing the moderating effects, we mean-centered the variables to avoid multicollinearity. As demonstrated in Model 3 of Table 5, the interaction term between ego depletion and pay satisfaction was positively related to compulsive buying (*b* = 0.125, *p* < 0.01), indicating that pay satisfaction moderates the relationship between ego depletion and compulsive buying. Furthermore, to facilitate interpretation, we plotted the interaction effects using simple slope analysis. As shown in Figure 2, the positive relationship between ego depletion and compulsive buying was stronger when pay satisfaction was high. The simple slope tests demonstrated that ego depletion was positively related to compulsive buying when pay satisfaction was low (*b* = 0.243, *p* < 0.001). This relationship was stronger when pay satisfaction was high (*b* = 0.554, *p* < 0.001). Thus, Hypothesis 4a was supported.

**TABLE 5:** Results of moderation analyses (*N* = 445).

Variables	Compulsive buying			Compulsive eating		
	Model 1	Model 2	Model 3	Model 4	Model 5	Model 6
Constant	4.275***	3.607***	3.787***	4.791***	3.637***	3.714***
Gender	0.252	0.222	0.203	0.216	0.168	0.160
Age	−0.163	−0.022	−0.045	−0.320***	−0.134	−0.144*
Education	0.088	0.064	0.073	−0.072	−0.087	−0.083
Monthly income	−0.27***	−0.188**	−0.181**	−0.210***	−0.046	−0.043
Ego depletion		0.355***	0.398***		0.392***	0.411***
Pay satisfaction		0.069	0.053		−0.142**	−0.149**
Ego depletion × pay satisfaction			0.125**			0.053
*R* ^2^	0.067	0.154	0.174	0.097	0.246	0.005
Δ*R*^2^	0.067	0.086	0.021	0.097	0.342	0.347
*F*	7.926***	13.239***	13.165***	11.783***	38.023***	33.238***

*p < 0.05,

**p < 0.01,

***p < 0.001.

**FIGURE 2:**
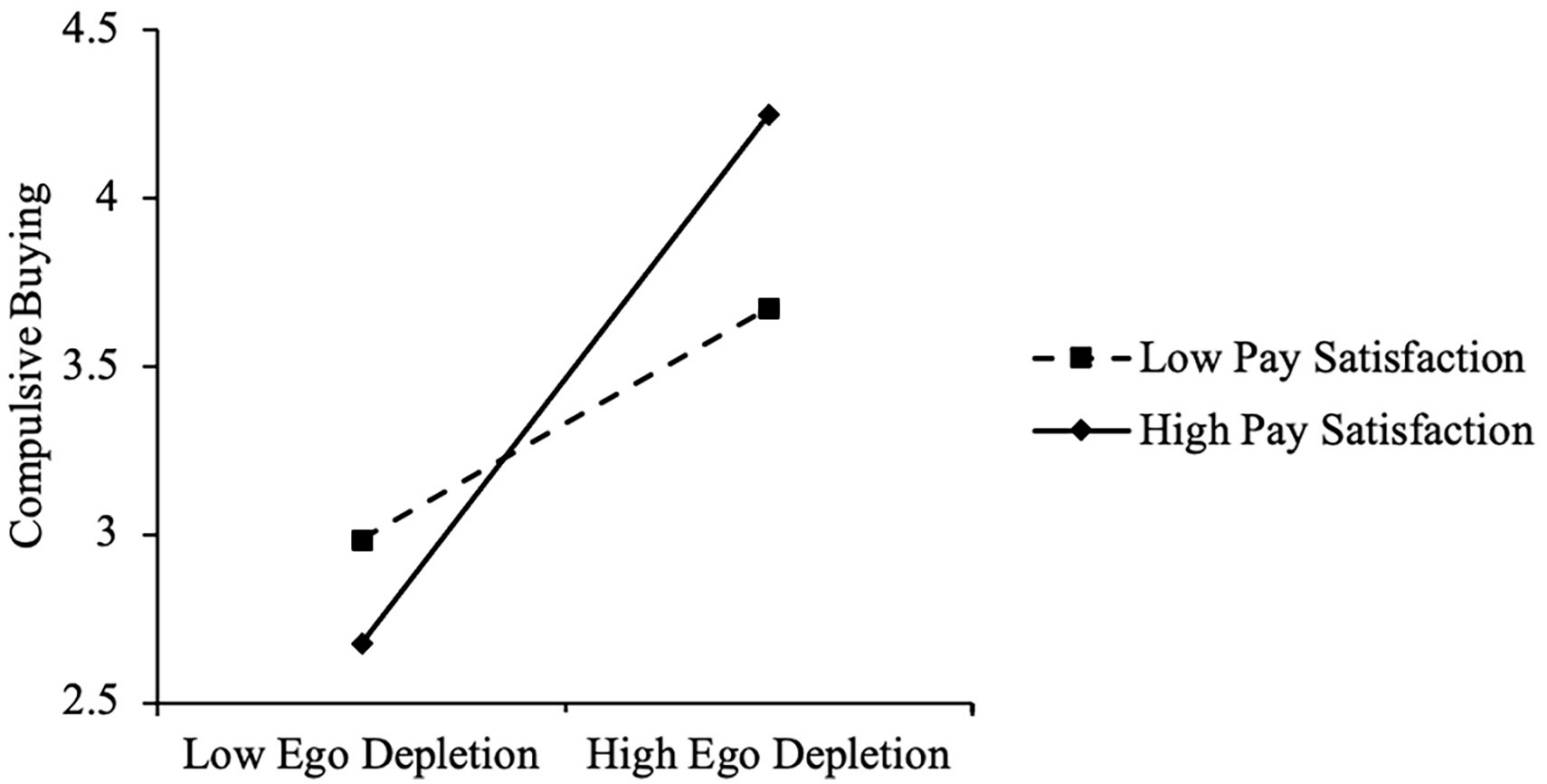
The moderating effect of pay satisfaction on the relationship between ego depletion and compulsive buying.

According to Model 6, the interaction term between ego depletion and pay satisfaction was not statistically significant in predicting compulsive eating (*b* = 0.053, *p* = 0.069). As shown in Figure 3, simple slope tests indicated that ego depletion was positively related to compulsive eating both when pay satisfaction was low (*b* = 0.344, *p* < 0.001) and when pay satisfaction was high (*b* = 0.477, *p* < 0.001). Although the association was numerically stronger at higher levels of pay satisfaction, the interaction effect did not reach the conventional level of statistical significance. Therefore, Hypothesis 4b was not supported.

**FIGURE 3:**
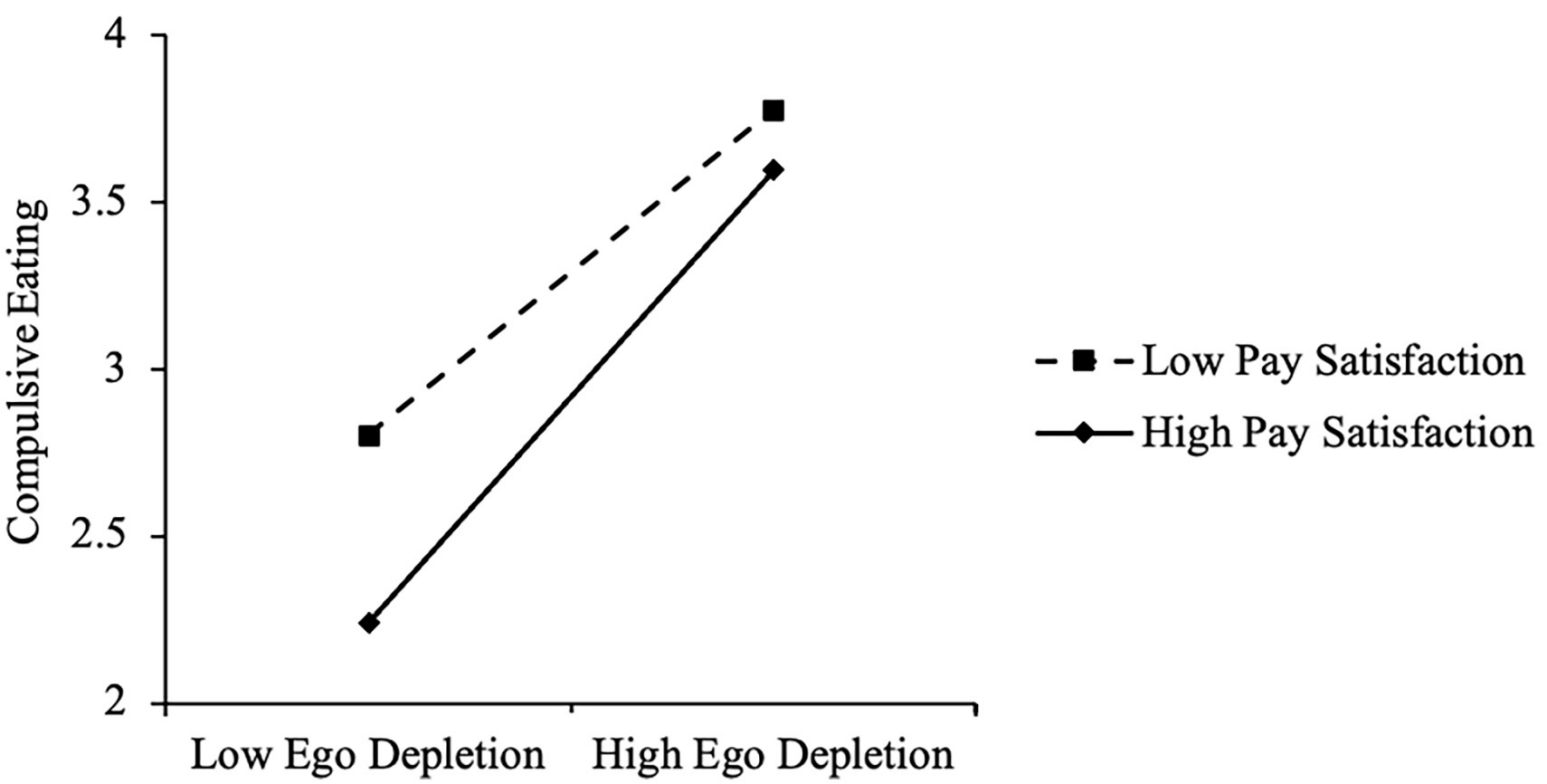
The moderating effect of pay satisfaction on the relationship between ego depletion and compulsive eating.

#### 4.3.4 Moderated mediation effects

To test the moderated mediation model, we conducted an integrated latent variable structural equation model analysis. The model was estimated using robust maximum likelihood estimation with Monte Carlo numerical integration. Gender, age, education level and monthly income were controlled in the structural model. As shown in Table 6, for compulsive buying, the conditional indirect effect of abusive supervision on compulsive buying via ego depletion was significant at low levels of pay satisfaction, indirect effect = 0.263, 95% CI [0.091, 0.436], at the mean level of pay satisfaction, indirect effect = 0.430, 95% CI [0.251, 0.608], and at high levels of pay satisfaction, indirect effect = 0.596, 95% CI [0.364, 0.829]. The index of moderated mediation was also significant, index = 0.144, 95% CI [0.057, 0.230], providing support for Hypothesis 5a.

**TABLE 6:** Integrated latent variable SEM results (*N* = 445).

Outcome	Moderator	Level	Estimate	95% CI	The index of moderated mediation
Compulsive buying	Pay satisfaction	Low	0.263	[0.091, 0.436]	0.144 95% CI [0.057, 0.230]
		Mean	0.430	[0.251, 0.608]	
		High	0.596	[0.364, 0.829]	
Compulsive eating	Pay satisfaction	Low	0.426	[0.237, 0.614]	0.037 95% CI [−0.040, 0.115]
		Mean	0.469	[0.299, 0.638]	
		High	0.512	[0.317, 0.706]	

High and low levels of the moderators represent values at +1SD and −1SD from the mean, respectively.

For compulsive eating, the conditional indirect effects were significant at low levels of pay satisfaction, indirect effect = 0.426, 95% CI [0.237, 0.614], at the mean level of pay satisfaction, indirect effect = 0.469, 95% CI [0.299, 0.638], and at high levels of pay satisfaction, indirect effect = 0.512, 95% CI [0.317, 0.706]. However, the index of moderated mediation was not significant, index = 0.037, 95% CI [−0.040, 0.115]. Thus, the integrated latent variable model indicated that pay satisfaction did not significantly moderate the indirect effect of abusive supervision on compulsive eating via ego depletion. Therefore, Hypothesis 5b was not supported.

## 5 Discussion

This study explores the relationship between abusive supervision, ego depletion, and compulsive consumption behaviors such as compulsive buying and compulsive eating, with an emphasis on the moderating role of pay satisfaction. By integrating these concepts, we shed light on the psychological mechanisms through which abusive supervision spills over into employees’ personal lives, providing both theoretical contributions and practical implications. Our findings offer valuable insights into the effects of workplace mistreatment, the role of ego depletion, and the influence of pay satisfaction on maladaptive behaviors.

### 5.1 Theoretical contributions

Our research makes several incremental theoretical contributions. First, this study advances the abusive supervision literature by offering a process-based explanation of how abusive supervision spills over into employees’ personal lives. Abusive supervision has been shown to spill over into the family domain through work-to-family conflict and family undermining, or to impair employees’ sleep and next-day well-being through insomnia and rumination (Fischer et al., 2021; Mackey et al., 2017; Tepper et al., 2017). Related research on workplace mistreatment and unhealthy consumption has also tended to explain nonwork outcomes through negative affect, strain, or resource depletion (Liu et al., 2017). While this literature has importantly demonstrated that abusive supervision and other work stressors can cross domain boundaries, it has paid less attention to the self-regulatory process through which such experiences shape specific off-job behaviors. Different from a traditional resource-exhaustion account, we draw on the contemporary process model of ego depletion to conceptualize depletion as shifts in motivation and attention rather than the mere loss of a finite resource (Friese et al., 2019; Inzlicht et al., 2014; Inzlicht and Schmeichel, 2012). From this perspective, abusive supervision may lead employees to suppress negative emotions, inhibit retaliatory impulses, and monitor interpersonal threats, thereby weakening their motivation for continued restraint and increasing their attention to immediately rewarding cues. This process-based perspective explains not only whether abusive supervision affects employees beyond work, but also why its spillover may take the form of compulsive buying and compulsive eating as consumption-related failures of self-regulation.

Second, our study modestly broadens the scope of abusive supervision outcomes by examining two forms of compulsive consumption: compulsive buying and compulsive eating. Prior research has documented that abusive supervision can spill over into employees’ personal lives in various ways (Bhattacharjee and Sarkar, 2024; Tepper et al., 2017), but relatively less attention has been paid to consumption-related self-regulatory failures. We focus on compulsive buying and compulsive eating because they are not simply additional examples of maladaptive nonwork outcomes; rather, they are especially relevant to the process model of ego depletion. Both involve recurrent difficulty resisting immediately rewarding consumption urges despite awareness of potential negative consequences, and both may serve short-term emotion-regulation or compensatory functions (Faber et al., 1995; Kakoschke et al., 2019; Ridgway et al., 2008). By examining these two outcomes, our study offers evidence that abusive supervision may be associated not only with broad strain or general spillover effects, but also with specific consumption-related manifestations of impaired self-regulation in employees’ private lives.

Third, our study contributes to the compulsive consumption literature by identifying abusive supervision as a workplace-related antecedent of compulsive buying and compulsive eating. Whereas prior research has focused largely on individual characteristics such as self-esteem, anxiety, stress, and low self-control (Sharma et al., 2026), our findings suggest that compulsive consumption is also shaped by employees’ day-to-day experiences with supervisors. Our study therefore shifts attention from intrapersonal vulnerabilities and generalized emotional states to a more distal but powerful interpersonal stressor in the workplace—abusive supervision (Darrat et al., 2016; Zheng et al., 2020). Thus, in line with the findings of Faber et al. (1995), our study verifies that compulsive buying and compulsive eating are not isolated consumer behaviors, but rather conceptualizes them as downstream consequences of workplace stressors operating through impaired self-regulatory functioning. Our findings broaden the nomological network of compulsive consumption and compulsive eating and responds to calls for more integrative explanations that connect emotional regulation and contextual stressors within a unified framework (Nanda et al., 2023; Tandon et al., 2021).

More importantly, our findings reveal a differential pattern between compulsive buying and compulsive eating, offering a key theoretical contribution to the abusive supervision spillover literature. Although both behaviors reflect consumption-related manifestations of impaired self-regulation, they appear to depend on different contextual conditions. Abusive supervision may create a general vulnerability to immediately rewarding coping behaviors by impairing employees’ self-regulatory functioning, but the specific form of spillover depends on whether the context matches the requirements of the behavior. Consistent with this view, pay satisfaction strengthened the relationship between ego depletion and compulsive buying, as well as the indirect effect of abusive supervision on compulsive buying through ego depletion, but did not significantly moderate the corresponding effects on compulsive eating. This pattern remained after controlling for monthly income, suggesting that the effect of pay satisfaction reflects not merely objective compensation but employees’ subjective sense of financial latitude. Because buying requires perceived economic latitude to translate depleted self-control into actual consumption, pay satisfaction may selectively amplify material coping mechanisms (Beatty and Ferrell, 1998; Hamilton et al., 2019). By contrast, compulsive eating may be more proximally shaped by somatic cravings, affect regulation, physiological cues, and habitual responses (Heatherton and Baumeister, 1991; Wood and Neal, 2007; Tiffany and Wray, 2012), making pay satisfaction a less behaviorally matched moderator. Thus, pay satisfaction should not be viewed as a general amplifier of compulsive consumption; rather, its moderating role depends on whether the financial latitude it represents is behaviorally matched to the outcome. In this sense, our study shows that the spillover effects of abusive supervision are not uniform across off-job compulsive behaviors, but depend on behavior-specific contextual contingencies.

### 5.2 Practical implications

The findings of this study have important practical implications for organizations, managers, and employees. First, organizations should recognize that the consequences of abusive supervision extend beyond work performance and may emerge in employees’ personal lives through unhealthy consumption behaviors, reduced well-being, and impaired self-regulation. Training programs aimed at increasing emotional intelligence, empathy, and conflict management skills among supervisors could reduce instances of abusive behavior and minimize the negative psychological impacts on employees (Haque and Lissillour, 2025). In addition, organizations should establish clear reporting channels and regular climate assessments to identify abusive supervisory practices before they escalate. Creating a supportive work environment in which employees feel safe to voice concerns may help reduce the spillover of workplace mistreatment into maladaptive off-job behaviors.

Second, organizations should recognize that favorable compensation alone may not fully offset the negative consequences of workplace mistreatment. Although pay satisfaction is generally associated with positive employee attitudes, our findings suggest that compensation practices should be complemented by efforts to maintain respectful supervisory relationships and employee well-being. Organizations should therefore avoid relying solely on financial rewards to address the consequences of stressful work environments and instead adopt a more holistic approach that integrates fair treatment, psychological support, and healthy work relationships.

Third, organizations can help employees maintain effective self-regulation when facing workplace stressors. Interventions such as emotional regulation training, mindfulness programs, and resilience-building workshops may help employees remain focused on long-term goals and reduce reliance on immediately rewarding but potentially harmful coping behaviors. By supporting employees’ capacity for adaptive self-regulation while simultaneously reducing workplace stressors such as abusive supervision, organizations can lower the likelihood that work-related strain spills over into maladaptive behaviors outside the workplace.

### 5.3 Limitations and future research directions

Despite its theoretical and practical contributions, this study is not without limitations. First, this study relied on self-reported data. This approach was appropriate given the private and difficult-to-observe nature of employees’ off-job consumption behaviors, but it may increase the risk of common method bias. Although we adopted a three-wave time-lagged design and implemented several procedural remedies, including temporal separation of measurements, anonymity assurances, and efforts to reduce evaluation apprehension, these procedures cannot fully eliminate common method variance or address all potential endogeneity concerns. Moreover, although the time-lagged design allowed us to examine the proposed spillover process across temporally separated waves, it was less able to capture short-term within-person fluctuations in abusive supervision, ego depletion, and consumption behaviors. Future research could therefore adopt multi-source, multi-method, or intensive longitudinal designs to further strengthen the robustness of these findings. For example, daily diary or experience sampling methods (ESM) would allow researchers to examine how day-to-day or moment-to-moment experiences of abusive supervision are associated with fluctuations in ego depletion and subsequent consumption behaviors in employees’ nonwork lives. Such designs would be particularly useful for capturing the dynamic and context-dependent nature of self-regulatory impairment and coping behaviors. In addition, incorporating objective or behavioral indicators of consumption, where feasible and ethically appropriate, could further reduce reliance on self-reports and provide stronger evidence regarding the spillover effects of workplace mistreatment on employees’ off-job consumption.

Second, while this study conceptualizes compulsive consumption as a maladaptive response to impaired self-regulation, the long-term consequences of this coping strategy remain unclear. Although compulsive buying and compulsive eating may provide temporary emotional relief, their repetitive and potentially addictive nature may ultimately generate additional psychological, financial, and health-related costs (DeSarbo and Edwards, 1996; O’Guinn and Faber, 1989; Ridgway et al., 2008; Vasiliu, 2022). Future research can adopt a longitudinal design to systematically examine whether these behaviors alleviate distress only in the short term while contributing to more severe maladaptive outcomes over time. Such research would help clarify the dynamic relationship between workplace stressors, self-regulatory functioning, and compulsive consumption behaviors.

Third, although this study examines compulsive buying and compulsive eating as two representative forms of compulsive consumption, future research could further explore other consumption-related spillover outcomes of abusive supervision, such as status consumption (Eastman et al., 1999), symbolic consumption (Berger and Heath, 2007), or hedonic consumption (Hur and Shin, 2025). More importantly, future research should move beyond identifying additional behavioral outcomes and examine whether different forms of consumption are driven by distinct psychological mechanisms. For example, while compulsive buying and compulsive eating may share a common basis in impaired self-regulation, other consumption behaviors may be more strongly driven by different processes, such as status signaling (Griskevicius et al., 2010), identity repair (Gollwitzer et al., 2013), or emotional compensation (Hirschman and Holbrook, 1986). Clarifying these mechanisms would help explain why abusive supervision leads some employees to engage in compulsive consumption, while others may respond through different forms of maladaptive or compensatory consumption. Such research would provide a more fine-grained understanding of how workplace mistreatment spills over into heterogeneous consumption behaviors in employees’ nonwork lives.

Finally, although our findings suggest that pay satisfaction strengthens the relationship between ego depletion and compulsive buying, our study does not directly measure the specific psychological processes through which pay satisfaction shapes employees’ consumption responses. We interpret pay satisfaction as a contextual condition that may reduce perceived financial constraints and make consumption opportunities more feasible when employees experience impaired self-regulation. However, we did not directly assess mechanisms such as perceived affordability, financial constraint, reward salience, or perceived consumption opportunity. Future research could examine these processes more directly to clarify why and when pay satisfaction shapes the translation of ego depletion into different forms of compulsive consumption.

## 6 Conclusion

This study provides a deeper understanding of the psychological processes linking abusive supervision to compulsive consumption behaviors, i.e., compulsive buying and compulsive eating. By highlighting the mediating role of ego depletion and the moderating role of pay satisfaction, our research expands the theoretical frameworks surrounding ego depletion, compulsive consumption, and abusive supervision. The findings offer both theoretical insights and practical guidance for organizations seeking to reduce the negative impact of abusive supervision and promote employee well-being.

## Data Availability

The raw data supporting the conclusions of this article will be made available by the authors, without undue reservation.
